# A qualitative internet-based study of parental experiences of adolescents suffering from affective disorders with non-suicidal self-injury during the COVID-19 pandemic

**DOI:** 10.3389/fpsyt.2024.1361144

**Published:** 2024-03-26

**Authors:** Yongna Wang, Xueqiu Chen, Chun Song, Yan Wu, Lihua Liu, Lili Yang, Xuege Hao

**Affiliations:** ^1^ Beijing Hui-Long-Guan Hospital, Peking University, Beijing, China; ^2^ Xinjiang Key Laboratory of Neurological Disorder Research, The Second Affiliated Hospital of Xinjiang Medical University, Urumqi, China

**Keywords:** non-suicidal self-injury, affective disorder, parental experience, adolescent, internet-based, qualitative research

## Abstract

**Objective:**

Non-suicidal self-injury (NSSI) behaviors of adolescents with affective disorders can directly deteriorate parents’ internal experiences, and negative parental experiences can exacerbate or even worsen NSSI behaviors. This study investigates the impact of NSSI behaviors exhibited by adolescents with affective disorders on the internal experiences of parents. Specifically, our research focuses on the inner experiences of parents when their children engage in NSSI behaviors during social isolation of the COVID-19, offering insights for addressing parental mental health issues related to NSSI and developing positive parental behavioral models to optimize adolescent behavior during major public health events.

**Methods:**

Semi-structured interviews were conducted with 21 parents of adolescents with affective disorders displaying NSSI behaviors during the COVID-19 pandemic. The Colaizzi 7-step analysis was employed to refine and categorize emerging themes.

**Results:**

Our study revealed that parents of adolescents facing NSSI during the COVID-19 pandemic underwent different internal experiences, which could be classified into four themes: negative experience, high caregiving burden, lack of caregiving capacity, and resilience.

**Conclusion:**

This Internet-based research is the first to explore the internal experiences of parents of adolescents with affective disorders experiencing NSSI during the COVID-19 pandemic. It sheds light on how parents, in response to their children’s NSSI behaviors, undergo resilience following negative experiences, explore more open and supportive family model. Despite these positive outcomes, parents express a need for increased knowledge about NSSI illness care and a desire for professional assistance.

## 1 Introduction

Non-suicidal self-injury (NSSI) is characterized by purposeful harm to body tissues through socially unacceptable means, such as cutting, skin burns, and impact, without any suicidal intent (1). This phenomenon is prevalent worldwide and consistently observed across different countries (2). In Chinese adolescents, the prevalence of NSSI is 22.37%, with a lifetime prevalence of 17.2%, which is 1.98 times higher than that observed in young adults. This rate further increases among adolescents with affective disorders like depression and bipolar disorder (3–5). Notably, during the COVID-19 induced social isolation, the incidence of NSSI behaviors in adolescents has experienced a significant surge (6, 7), exacerbating the mental state of adolescents with affective disorders and contributing to severe suicidal ideation (8, 9). NSSI has therefore become a major public health problem that requires urgent attention and a theoretical foundation needs to provide effective guidance of a solution to the kind of problem for future during similar public health events.

Understanding the internal experiences of parents of adolescents with NSSI is crucial for early detection and intervention, ultimately improving the well-being and long-term quality of life for adolescents with affective disorders (10). While existing research has predominantly focused on exploring the characteristics, causal mechanisms, risk factors, and intervention strategies for adolescents with NSSI (11–14), and some studies have investigated the emotional experiences of adolescents engaged in NSSI (15, 16), there remains a notable scarcity in research addressing the emotional reactions and experiences of parents in this context.

Adolescents’ NSSI behaviors often disrupt normal family life, shifting it toward a “patient-centered” mode of functioning, making it challenging for parents to maintain their daily lifestyle and leading to diverse mental health problems (17, 18). Parents play a crucial role as behavioral models for adolescents during the critical period of character formation. The psychological problems and behavioral patterns of both parents and adolescents are interdependent and mutually influential (19).. On the one hand, parents, as a vital part of the social support system for adolescents with affective disorders, bear the burden of long-term treatment and rehabilitation, coupled with economic, work, and social pressures, resulting in varying degrees of psychological problems and adversely affecting their quality of life (20, 21). Simultaneously, the context of social isolation due to the COVID-19 epidemic has heightened NSSI incidence among adolescents, leading to recurring illnesses and further burdening parents with care responsibilities. Following the ecosystem theory, the family serves as the largest microsystem, with parents influencing adolescents’ emotional cognition (22).Parents’ experiences and external information feedback directly impact offspring, shaping their behavioral styles. Negative emotional reactions from parents hinder the prognosis and recovery of NSSI adolescents, exacerbate family burdens, and create a vicious circle (23–25). Thus, focusing on parents’ internal experiences of NSSI behaviors in adolescents with affective disorders holds significant importance in shaping positive parental behavior models and promoting the optimal functioning of the family system for NSSI adolescents.

This study conducted in-depth Internet-based interviews with parents of adolescents with affective disorders associated with NSSI. The aim was to gain insights into the internal experiences of these parents while caring for their adolescents with NSSI during the social isolation of the COVID-19 pandemic. This research provides valuable information for future early responses to mental health problems of parents with NSSI in the context of similar major public health events. Additionally, the study seeks to offer theoretical references for the future development of family interventions, enhancing parent-child interaction styles between parents and adolescents with NSSI and shaping positive parental behavioral models to optimize adolescent behavioral patterns.

## 2 Materials and methods

### 2.1 Study setting

This study was a cross-sectional survey conducted in a tertiary psychiatric specialized hospital in Beijing.

Prior to the semi-structured interview (SSI), informed consent was obtained from the adolescents and their parents. The study protocol received approval from the Ethics Committee of the Beijing Hui-Long-Guan Hospital (LY202210).

### 2.2 Participants

Participants in this study consisted of parents of 21 inpatients (5 boys and 16 girls) diagnosed with affective disorders accompanied by NSSI behaviors. The recruitment period spanned from September to November 2022, and the study took place at Beijing Hui-Long-Guan Hospital.

Adolescent patient inclusion criteria were as follows: (a) aged between 10 and 19 years; (b) meeting the diagnostic criteria for non-suicidal self-injury as defined in the Diagnostic and Statistical Manual of Mental Disorders, Fifth Edition (DSM-5), experiencing self-injury at least 5 days during the preceding 12 months without any suicidal intention (26); (c) meeting the diagnostic criteria for affective disorders according to the International Classification of Diseases (ICD-10) Classification of Mental and Behavioral Disorders: Clinical Descriptions and Diagnostic Guidelines (27). Exclusion criteria for patients were: (a) the presence of severe physical illnesses; (b) co-existing other mental disorders. For parents, inclusion criteria comprised: (a) selecting parents aged between 35 and 60 to mitigate the effects of aging, hormonal fluctuations, and other physiological changes on physical and mental states (28, 29). (b) having a close relationship with the patient, defined as serving as their primary caregiver and providing a minimum of four hours of care per week in the past 3 months (30). (c) possessing the ability to communicate and express themselves verbally without serious physical or mental illnesses; (d) having regular economic income; (e) lacking religious beliefs; (f) not having a COVID-19 infection. Exclusion criteria for parents were: (a) the presence of mental disorders; (b) significant life changes within the past six months, such as the loss of close relatives or divorce; (c) being in single-parent families, as this may lead to higher negative parental experiences than regular families. Adolescents and parents signed an informed consent form before the start of the study.

### 2.3 Interview procedures

The interviews were conducted by a Ph.D. researcher with a decade of clinical experience in counseling. Additionally, two Master of Nursing students with clinical experience provided support during the interviews. Before initiating the study, each researcher underwent comprehensive training on the study protocol and human subjects research, emphasizing aspects such as privacy protection and respect. The principal investigator, who possessed full knowledge of the patients’ conditions due to involvement in their clinical care, provided training to the researchers based on the patients’ medical records. The researchers also underwent a 5-day training program covering qualitative research, interview guidelines, communication skills, and emergency response. This training was documented in a portable handbook for ongoing reference and updates.

SSIs with patients were conducted using phenomenological research methods, with each interview lasting approximately 30 minutes. The interviews took place between September 2022 and November 2022.

The interview framework, designed to align with the research objectives, was developed through literature analysis and consultations with experts, including two registered psychiatrists with doctoral degree, six psychiatric nursing managers, and six psychiatric nurses with over a decade of experience in adolescent mental disorder units.

Pre-interviews with two respondents were conducted to refine and revise the framework. The finalized interview outline included key questions such as: (a) How did you first become aware of your child’s self-injurious behavior? (b) What were your emotions when you realized that your child was engaging in self-injury? (c) How do you perceive your child’s self-injurious behavior, and what do you believe are the underlying reasons for it? (d) What has been the impact on both you and your family? (e) Have there been instances that you preferred to keep hidden or deliberately concealed from others? (f) Currently, what is your most pressing concern, and what kind of assistance do you wish to receive?

Due to the impact of COVID-19, all interviews were uniformly conducted through online video platforms. Prior to the interviews, the interviewer informed participants about the purpose and significance, assuring strict adherence to confidentiality principles. To protect the participants’ privacy, their names were replaced with the letters A-U. Throughout the interviews, the researchers closely observed changes in the subjects’ facial expressions, intonation, and emotional cues. They were encouraged to openly express their feelings, and various interview techniques, including questioning, counter-questioning, and clarification, were employed when necessary. With the consent of the interviewees, the entire interview was recorded in real-time, and subsequently, the interviewer transcribed the interview for further analysis.

### 2.4 Data analysis

The qualitative data analysis software NVivo was employed for importing and analyzing the data in this study. Colaizzi’s method of data analysis, rooted in Husserl’s phenomenological philosophical positions and utilizing seven steps, was utilized to critically examine the textual record and provide an in-depth description of the phenomenon under investigation (31, 32). Initially, the interviewer and another researcher maintained a neutral on-site interview diary for each interview, recording primarily non-verbal behaviors of the participants. Subsequently, the audio-recorded data were transcribed into written text 24 hours after the interviews, incorporating the non-verbal content from the diary, ensuring careful cross-checking for accuracy. The second step involved identifying statements related to “parents’ internal experiences about their adolescent with NSSI”. Third, recurring and meaningful ideas were coded during repeated readings, forming an initial coding framework. This framework was scrutinized by the entire group to mitigate the risk of bias. In the fourth step, the researchers organized the initial codes into “rough” themes based on frequencies and similarities in the data. The “rough” themes were continually refined and sequenced through review and examination to ensure hierarchy, coherence, and logic. The fifth step involved developing a thematic structure by comprehensively describing and synthesizing parents’ internal experiences of their NSSI adolescents. In the sixth step, all researchers revisited the thematic structure to further refine the themes. Finally, the shaped thematic clusters were returned to the respondents via email to verify the authenticity and accuracy of the themes. Throughout the data collation and analysis process, the results were continuously reviewed and discussed by all researchers to reach a consensus. This iterative approach, rather than a linear one, ensured the reliability of the results.

## 3 Results

The sociodemographic and clinical characteristics of adolescent patients and interviewees are presented in **Table 1**.

**Table 1:** Characteristics of participants (N=21).

Socio-demographic Characteristic	Number
*Age of Parents (Mean, Standard Deviation)*	42.6,16.0
Gender of Parents	
Female	16(76%)
Male	5 (24%)
Education Background of Parents	
Less than High School	8(38%)
High School	8(38%)
More than High School	5(24%)
*Age of Patients*	15.3,3.1
Gender of Patients	
Girl	16(76%)
Boy	5 (24%)
Diagnosis of Patient	
Depressive Disorders	15(71%)
Bipolar Disorders	6 (29%)

Analysis of the interview data resulted in 4 themes and 13 subthemes, as presented in **Table 2**.

**Table 2:** The parental experiences of adolescents with NSSI during the COVID-19.

Themes	Subthemes
*Negative Experience*	Shocking and Helplessness
	Guilty
	Dilemma
*High Caregiving Burden*	Uncertainty
	Financial Burden
	Stigma
*Lack of Caregiving Capacity*	Misunderstanding
	Ineffective Coping
	High Expectations
	Craving Assistance
*Resilience*	Reflection
	Intimacy
	Prioritize Mental Health

### 3.1 Negative experience

#### 3.1.1 Shocking and helplessness

Parents expressed profound shock, distress, and fear upon discovering their child’s self-injurious behavior for the first time. As the behavior recurred, feelings of frustration and helplessness intensified.

*“She tries to injure herself whenever she is under stress … her resilience is too low … It’s so helpless….” Participant E.*

*“We don’t dare to give her any pressure because she is sick, but she still hurts herself from time to time … it’s really hopeless (frown).” Participant F.*

#### 3.1.2 Guilty

With an increasing awareness of adolescent mental illness and a recognition of the significant impact of family factors, some parents attributed their child’s illness to themselves, resulting in self-blame and guilt.

*“I do feel quite bad, blaming myself from the bottom of my heart that I spend too little time with her and my child is too lonely (choking).” Participant A.*

*“I pushed her to study and put too much pressure on her, so I can’t shirk my responsibility for my child’s illness.” Participant D.*

*“The main reason for the child being like this lies in us as parents. Her father and I often quarrel and have cold wars. Before, I always thought that since she was little, she didn’t understand this. But now it seems the effect on her has been dire.” Participant H.*

#### 3.1.3 Dilemma

In this study, many participants believed that one of the most important reasons for adolescents’ self-injurious behaviors was the “pressure of studying” and that parents would deliberately reduce the pressure of studying but would not want to lower their long-term expectations of their children.

*“On the one hand, I want her to do well in her studies and haven’t given up my expectations for her, but on the other hand, I don’t want her to be hurt because of the pressure I put on her. I get anxious when her grades come down and enroll her in all kinds of classes, and when she can’t take it anymore, I’ll stop it again … I’m torn…” Participant D.*

*“She is now sick and has had to take some time off school. When she is feeling a little better, I still want to encourage her to push herself to keep up with her classes.” Participant J.*

Parents are becoming aware of the importance of maintaining the physical and mental health of adolescents for their overall development, and are taking measures to try to reduce the pressure on their children to attend school. However, once parents consider their children’s future survival, they are filled with worry and display a complex and contradictory emotion.

*“I also know that she is under great pressure to study, but if she doesn’t study, what will she do in the future? Her parents can’t support her for the rest of her life.” Participant P.*

*“If we don’t let her go to school now, she will be much better off, but what will she do in the future? If she doesn’t go to university, how will she find a job? She will blame us when she grows up.” Participant R.*

*“I don’t dare to push her now, it doesn’t matter if she’s good or bad at school, my daughter’s life is important, but thinking about her future makes people worry…” Participant T.*

### 3.2 High caregiving burden

#### 3.2.1 Uncertainty

Parents experienced high psychological pressure due to the emotional volatility of adolescent patients and the repeated occurrence of self-injurious behaviors. Uncertainty about the prognosis of the disease and the effectiveness of treatment further adds to the psychological burden of parents.

*“…I don’t dare to leave her alone for fear that something will happen to her. I’m under a lot of pressure (sigh).” Participant I.*

*“Is this kind of mental illness something that can’t be cured once you have it? Her mother and I are especially worried about this…” Participant Q.*

#### 3.2.2 Financial burden

The recurrence of illnesses placed a substantial financial burden on families, including treatment costs and compensatory spending on travel, shopping, and entertainment.

*“Seeing a doctor costs money, and I have to work hard to earn money and save money, and my friends say that I have emaciated a lot, which can’t be helped.” Participant B.*

*“She says she doesn’t feel secure with less pocket money, so I don’t dare control her spending, and she spends much money on shopping and going out to play, so we can barely afford to pay for it.” Participant U.*

#### 3.2.3 Stigma

Due to the lack of public knowledge about psychiatric illnesses, some parents are concerned about the stigma attached to the illness, thus creating a sense of collateral stigma.

*“When friends ask me about my child, I keep it to myself … people don’t know much about it, and they don’t know what to think when they hear about the disease…” Participant A.*

*“I would tell her to hide those scars when we go out; people look at us strangely and point…. We rarely go out anymore….” Participant H.*

Other parents said that adolescent illnesses put psychological and social pressure on them, and that they would fear social scrutiny, and therefore would not dare to reveal the true situation of their families, and would be forced to cut down on socializing.

*“Because of my child’s illness, we moved, and we don’t communicate with our old neighbors anymore. I’m afraid that they will always ask me about my child, and I don’t know what to say…” Participant E.*

*“Rarely go to class reunions anymore, you are no more successful at work, and life is a failure when you have a child at home who often self-harms.” Participant O.*

*“I’m quite facetious; I don’t want to let others know the worst side of my family.” Participant S.*

### 3.3 Lack of caregiving capacity

#### 3.3.1 Misunderstanding

Parents exhibited a lack of understanding about NSSI and struggled to interpret their child’s self-injurious behaviors correctly.

*“I don’t understand the child’s self-injury. I don’t know if it’s because my remarriage is affecting her or if it’s because she wants something…” Participant F.*

*“I don’t know what’s going on in his mind, why he’s hurting himself like this…” Participant K.*

*“I particularly don’t understand why he’s like this. Is he running away from something?” Participant G.*

In addition, parents expressed their inability to understand or even suspicion that the adolescent’s self-injurious behavior was a sign of inanity or a period of rebellion, and parents expressed strong dissatisfaction with this.

*“She has everything she wants and always says she’s condescending. I don’t know what she’s aggrieved about. Sometimes I think she’s just picking on things for no reason.” Participant E.*

*“… She’s just threatening me, it’s pissing me off…” Participant H.*

*“When a child reaches the rebellious stage, frankly speaking, he’s just looking for trouble!” Participant N.*

#### 3.3.2 Ineffective coping

Parents generally lacked the ability to identify and cope with self-injurious behaviors triggered by adolescents’ psychological problems.

*“What am I going to do if she starts getting irritable again and wants to hurt herself?” Participant H.*

*“I don’t know when she’s in one of those bad moods; sometimes, I’m baffled when she hits herself.” Participant U.*

Some parents choose avoidant coping styles when caring for a young person with NSSI because they fear that talking about certain sensitive topics may trigger resistance or emotional stress in their child.

*“Sometimes she takes the initiative to talk to me about self-injurious behaviors, and I don’t know how to answer, so I can only change the topic in a hurry.” Participant A.*

*“The doctor said she meets the criteria for discharge, but I still don’t dare to pick her up and bring her back, I don’t know how to take care of her…” Participant D.*

#### 3.3.3 High expectations

Some parents expressed dissatisfaction with the lengthy treatment programs and the lack of care for their children due to inadequate staffing.

*“It’s been months since the treatment, and it’s still not complete; I don’t know how much longer it will take…” Participant B.*

*“I brought her to a big hospital for treatment because I want to get to the root of the disease and get her well in a hurry.” Participant I.*

*“Doctors and nurses should care more about and look after the child and give her enough love. she will surely get well quickly.” Participant T.*

Lack of knowledge about the disease makes it difficult for parents to understand the complexity of adolescent NSSI behaviors, leading to high expectations of treatment outcomes and a desperate desire for their child to be cured.

*“She doesn’t really cooperate with psychotherapy. Can’t she get better if she takes medication alone?” Participant C.*

*“I think with a little more treatment, she will be back to normal, I am very confident…” Participant P.*

*“Nowadays medicine is so advanced, and there are so many treatment methods, it shouldn’t be difficult to cure her…” Participant R.*

#### 3.3.4 Craving assistance

Respondents expressed a desire for professional help to better understand and cope with their adolescent patients and prevent the recurrence of the disease.

*“Those methods of communicating with children that the nurses taught me before were particularly good, and I still consult them when I encounter problems.” Participant M.*

*“I think there should be some initiatives at the national level for some professionals to publicize and popularize the knowledge of adolescent mental health.” Participant S.*

### 3.4 Resilience

#### 3.4.1 Reflection

In caring for adolescents with NSSI, some parents not only demonstrated sustained self-reflection, but also initiated positive role adjustments, a process that not only enriched their caregiving experience, but also fostered positive, healthy patterns of family interactions, which allowed for a profound experience of personal growth.

*“Because I am afraid that my child will be affected, I will now deliberately avoid quarreling with her father, and peace of mind can also solve problems.” Participant H.*

*“Thinking about myself, I do have problems; I am working on them, and my child even praised me on the phone yesterday.” Participant J.*

Some parents reported a positive change in their mindset to focus more on building a close relationship with their children, and this change had a positive impact on alleviating the anxiety of their teenagers.

*“In the past, I always thought of earning more money for her to spend, but now I realize that my daughter doesn’t need money; she needs her mother, and now that I am with her a lot, she’s much happier (laughs).” Participant C.*

*“In the past, I was too perfectionist. My son was greatly influenced by me. Slowly, after I became less strict, he didn’t get so tense and anxious.” Participant G.*

#### 3.4.2 Intimacy

Despite the heavy impact of a teenager’s illness on the family, the process of coping together fostered closeness among family members who care for and support each other.

*“My mother-in-law, who used to be quite firm with me, surprised me this time. In order to support her granddaughter’s medical treatment, she handed over her bankbook. I found it quite touching.” Participant D.*

*“Her father used to yell at her, and she wasn’t close to him at all. But now I see the two of them playing together a lot more. Their relationship has improved tremendously.” Participant I.*

*“My husband didn’t used to stay at home much., but after the child’s illness, he has changed a lot and took the initiative to take over all the housework.” “Thinking about myself, I do have problems; I am changing them, and my child even praised me on the phone yesterday.” Participant J.*

#### 3.4.3 Prioritize mental health

Firstly, some parents reported becoming concerned about mental health and learning from others about their own experiences of care in the hope of encouraging other parents to pay attention to their child’s potential mental health problems.

*“… I know now that not only the body is important, but also the mind…” Participant K.*

*“I often share information about depression with my friends. By drawing on my personal experience, I want to encourage other parents to pay attention to potential mental health issues in their children. “ Participant E.*

Secondly, some parents are becoming aware of their individual mental health problems and are beginning to take positive steps to adjust. This change helped them to understand their child’s behavioral patterns and had a positive effect on their own mental health.

*“In order to solve some doubts, I have done psychological counseling more than ten times, and now I can understand my child.” Participant G.*

*“Because my child got sick, I started to listen to some positive psychology courses and slowly adjusted myself; my temper is not so quick, and I am not so controlling (laughs).” Participant H.*

## 4 Discussion

To the best of our knowledge, no study has hitherto conducted a qualitative analysis of the parental experience of adolescents with affective disorders accompanied by NSSI during a major global public health event through online platforms. A pivotal discovery from this investigation is the improvement of negative family living patterns and increased family resilience as parents collaborate with their adolescents to address NSSI, a facet not previously documented in the literature. The second finding was that parents of adolescents with NSSI in this study lacked knowledge about illness care, exacerbating the burden of care. Additionally, this study found that parents conflicted reducing academic stress and maintaining high expectations among adolescents with NSSI due to the high value placed on educational achievement and traditional values.

Family communication theory underscores communication within the family as a feedback loop process, where supportive behaviors and language foster positive feedback, aiding family members in establishing and sustaining close relationships. This process improves the family’s ability to resolve internal conflicts and external stressors, creating a virtuous cycle (33). Past research has indicated that adolescents’ NSSI behaviors often lead to an escalation in controlling behaviors by parents, driven by a desperation for their children to adhere to guidance for positive adjustments. However, such efforts typically prove counterproductive and increase NSSI frequency (34, 35). In contrast, the parents in this study, after experiencing shock, guilt, and helplessness, engaged in a re-evaluation of their prior parenting styles. They actively adjusted their interaction approaches with their adolescents experiencing NSSI, incorporating supportive parenting behaviors such as companionship, empathy, and avoidance of family conflict. This positive shift contributed to the enhancement of parent-child relationships and the cultivation of a more harmonious family atmosphere. Simultaneously, family members joined forces in caring for and supporting adolescents with NSSI, fostering increased family intimacy and improved family functioning. Several studies have reported that enhanced family functioning significantly reduces adolescents’ suicidal ideation and behavior, with the treatment effects enduring for up to 6 months (34, 36, 37). Consequently, future longitudinal studies should explore the impact of parents adjusting their interaction styles with their children on adolescents’ symptomatology and NSSI frequency. Furthermore, this study revealed that, post-adjustment, most parents exhibited heightened concerns about mental health. They actively collaborated with their children to acquire emotion management skills and reinforce family coping strategies for dealing with negative events. Thus, the current study substantiated that a harmonious family atmosphere contributes to a reciprocal parent-child binary relationship model, enhances stress-coping skills within the family, strengthens the protective support of the family system for adolescents, and promotes mental health and overall regression of NSSI in adolescents (36).

The second major finding of the study indicates that parents not only struggle to cope with their adolescents’ NSSI behaviors but also bear a diverse burden of care. This phenomenon is partly attributed to the severe shortage of mental health personnel in China and the lack of comprehensive education on mental health knowledge (30). Consequently, families dealing with mental illness lack essential mental health knowledge, leading to a low level of parental awareness of NSSI. Parents find themselves unable to provide psychological support and care when adolescents experience NSSI behaviors. Moreover, they hold unrealistically high expectations regarding the effectiveness of treatments for adolescent NSSI, anticipating swift eradication of affective disorders and associated NSSI behaviors (30, 38). However, adolescent NSSI behaviors are often recurrent, addictive, and insidious, resulting in problems such as serious infections and disabilities. This escalates treatment costs, exacerbates family financial pressures, and ultimately leads to parental dissatisfaction with treatment outcomes (39). On the other hand, Confucianism, dominant in guiding social behavior in China, may view the symptoms of mental illness as a violation of Confucian principles of social order and harmony. Families experiencing mental illness may be labeled as “morally deficient”, leading to a sense of “lose face” for the patient and family members (40, 41). Consequently, parents feel ashamed to discuss their children’s NSSI behaviors with outsiders, resulting in social withdrawal and further increasing the burden of care on parents. This underscores the urgency of enhancing public and family mental health knowledge to more effectively support and manage families dealing with adolescent NSSI.

Finally, parents in this study experienced various negative psychological challenges in parenting NSSI adolescents, with a significant internal conflict arising from parents’ attempts to reduce their children’s schooling stress while being unwilling to diminish their long-term expectations for their children. In traditional Chinese culture, education plays a crucial role in improving one’s character and social status. Parents are willing to invest considerable time and resources to ensure their children receive a high-quality education and stand out in the fierce social competition, enhancing family prestige and social status (42). Even when their children exhibit serious NSSI behaviors, parents still hope that NSSI adolescents can quickly correct their mentality and focus on their studies, aiming to keep up with or surpass their peers’ academic progress. This tension in parent-child relationships exacerbates adolescents’ psychological pressure and may even lead to an increase in NSSI behaviors, potentially triggering serious suicidal ideation and behaviors (18, 43). Thus, the present study emphasized the importance of understanding and addressing this internal conflict when providing support to adolescents with NSSI and their families, facilitating the development of more effective intervention and support strategies.

Our findings suggest that future efforts should concentrate on enhancing parents’ ability to recognize the positive aspects of dealing with the disease to maintain a well-functioning family system for adolescents with NSSI. Existing research proposes that dialectical behavior therapy (DBT) is an effective approach for broadening parental perspectives by promoting balanced and multiple viewpoints. This can mitigate extreme thinking among parents and NSSI adolescents, offering more balanced solutions for NSSI families (14). Secondly, diverse methods of disseminating disease knowledge should be actively explored, such as Internet-based self-help psychological counseling services, to address the shortage of mental health resources (44). Simultaneously, medical social workers should raise awareness about the assistance policies provided by local governments for individuals with mental illnesses. They should encourage parents to actively apply for and benefit from these policies, thereby reducing the financial burden on their families. Furthermore, advocating for the “hospital-school-family” tripartite cooperation mechanism is crucial. Collaboratively formulating a plan for adolescents to return to school can facilitate their successful completion of education and alleviate parental concerns about their children’s future (45). These integrated interventions aim to promote the coordinated functioning of the family system, enhance support for adolescents, integrate them better into school and society, and alleviate parental concerns.

This pioneering internet-based study examines the internal experiences of parents with affective-disordered adolescents displaying NSSI during COVID-19 and offers timely insights on enhancing family resilience while addressing parental mental health concerns amidst global public health crises. However, this study has some limitations that should be acknowledged. First, although the gender of each NSSI adolescent’s participating parent differed due to traditional Chinese culture, with females typically devoting more time to caring for adolescents with NSSI (18), there were also fathers caring for the patients in this study. Consequently, despite the difference in the gender proportion of the interviewees, those included were fully aware of and caregivers for the affected children. Future studies plan to include both parents to explore family interaction patterns and the dilemmas faced by adolescents with NSSI more comprehensively. Second, this study predominantly involved female NSSI adolescents, aligning with the higher incidence of NSSI in female Asian adolescents compared to males (19). As a result, the findings are more relevant to the emotional experiences of parents of female NSSI adolescents. Finally, although this study employed internet-based semi-structured interviews, lacking face-to-face interaction for complete observation of physical movements and subconscious reactions, it still provides valuable evidence, especially considering the state of socially isolated major public health events. It may serve as a model for similar scenarios in the future.

## 5 Conclusion

This Internet-based qualitative study explored parental experiences of adolescents with NSSI during the COVID-19 epidemic. The findings revealed that parents underwent resilience, established innovative and positive family coping models, enhanced family resilience, and expressed a need for professional assistance despite a lack of knowledge about caring for adolescents with NSSI.

In the future, healthcare teams should prioritize providing specialized knowledge on illness care. This will empower parents of adolescents with NSSI to promptly adopt positive family coping models, ultimately contributing to the improvement of family resilience.

## Data availability statement

The raw data supporting the conclusions of this article will be made available by the authors, without undue reservation.

## Ethics statement

The studies involving humans were approved by Ethics Committee of the Beijing Hui-Long-Guan Hospital. The studies were conducted in accordance with the local legislation and institutional requirements. Written informed consent for participation in this study was provided by the participants’ legal guardians/next of kin.

## Author contributions

YoW: Software, Writing – review & editing, Writing – original draft, Supervision, Resources, Investigation, Data curation. XC: Software, Writing – review & editing, Writing – original draft, Methodology, Investigation, Data curation. CS: Writing – review & editing, Writing – original draft, Supervision, Methodology, Funding acquisition. YaW: Writing – review & editing, Supervision, Resources, Funding acquisition. LL: Writing – review & editing, Investigation, Data curation. LY: Writing – review & editing, Supervision, Investigation. XH: Writing – review & editing, Data curation.
